# Structure of a type IV CRISPR-Cas ribonucleoprotein complex

**DOI:** 10.1016/j.isci.2021.102201

**Published:** 2021-02-17

**Authors:** Yi Zhou, Jack P.K. Bravo, Hannah N. Taylor, Jurre A. Steens, Ryan N. Jackson, Raymond H.J. Staals, David W. Taylor

**Affiliations:** 1 Department of Molecular Biosciences, University of Texas at Austin, Austin, TX, USA; 2 Department of Chemistry and Biochemistry, Utah State University, Logan, UT, USA; 3 Laboratory of Microbiology, Wageningen University and Research, The Netherlands; 4 Institute for Cell and Molecular Biology, University of Texas at Austin, Austin, TX, USA; 5 Center for Systems and Synthetic Biology, University of Texas at Austin, Austin, TX, USA; 6 LIVESTRONG Cancer Institutes, Dell Medical School, Austin, TX, USA

**Keywords:** Biological Sciences, Structural Biology

## Abstract

We reveal the cryo-electron microscopy structure of a type IV-B CRISPR ribonucleoprotein (RNP) complex (Csf) at 3.9-Å resolution. The complex best resembles the type III-A CRISPR Csm effector complex, consisting of a Cas7-like (Csf2) filament intertwined with a small subunit (Cas11) filament, but the complex lacks subunits for RNA processing and target DNA cleavage. Surprisingly, instead of assembling around a CRISPR-derived RNA (crRNA), the complex assembles upon heterogeneous RNA of a regular length arranged in a pseudo-A-form configuration. These findings provide a high-resolution glimpse into the assembly and function of enigmatic type IV CRISPR systems, expanding our understanding of class I CRISPR-Cas system architecture, and suggesting a function for type IV-B RNPs that may be distinct from other class 1 CRISPR-associated systems.

## Highlights

• The type IV-B CRISPR-Cas Csf proteins assemble around an RNA
• First structure of a type IV-B CRISPR-Cas RNP complex
• Structure confirms evolutionary link of type IV systems from a type III-like ancestor
• Assembly of RNP complex on non-CRISPR RNAs suggests a non-canonical role

## Graphical abstract

**Figure:**
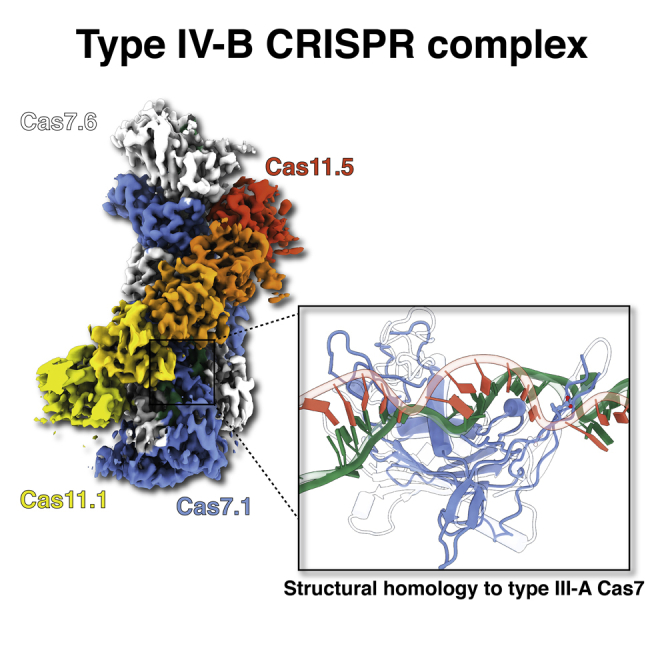

## Introduction

Bacteria and archaea employ CRISPR (Clustered Regularly Interspaced Short Palindromic Repeat)-Cas (CRISPR-associated) systems for adaptive immunity against phages, plasmids and other mobile-genetic elements (Makarova et al., 2020). In the multi-subunit class 1 systems, the CRISPR locus is transcribed and processed into small crRNA guides (CRISPR-derived RNA), around which several Cas proteins assemble to form large ribonucleoprotein (RNP) complexes that facilitate RNA-guided surveillance and degradation of complementary targets (Hille et al., 2018). While a myriad of structures have been determined for most types of CRISPR RNA-guided complexes (types I (Chowdhury et al., 2017; Jackson et al., 2014; Mulepati et al., 2014; Rollins et al., 2019; Xiao et al., 2018), II (Jiang et al., 2016; Jinek et al., 2014; Zhu et al., 2019), III (Jia et al., 2019; Sofos et al., 2020; Taylor et al., 2015; You et al., 2019), V (Li et al., 2021; Liu et al., 2019; Stella et al., 2017; Takeda et al., 2021; Zhang et al., 2020), and VI (Meeske et al., 2020; Slaymaker et al., 2019; Yan et al., 2018)), the RNP complexes of the highly diverse type IV CRISPR systems have largely remained structurally uncharacterized (Crowley et al., 2019; Faure et al., 2019; Makarova et al., 2020; Özcan et al., 2019; Taylor et al., 2019).

Type IV CRISPR systems primarily occur within plasmid-like elements, lack genes encoding adaptation modules (*cas1*, *cas2,* and *cas4*), and are classified into three distinct subtypes (IV-A, IV-B, IV-C) (Makarova et al., 2020; Özcan et al., 2019; Pinilla-Redondo et al., 2019). All type IV systems contain genes that encode for Csf2 (Cas7), Csf3 (Cas5), and Csf1 (large subunit) proteins, which assemble around an RNA to form a multi-subunit complex (Makarova et al., 2020; Özcan et al., 2019; Pinilla-Redondo et al., 2019). However, subtype-specific signature genes suggest distinct subtype functions. Type IV-A systems encode a DinG helicase shown to be essential for type IV-A mediated plasmid clearance (Crowley et al., 2019), Type IV-B systems contain the ancillary gene *cysH* of the phosphoadenosine phosphosulfate reductase family, and type IV-C systems encode a large subunit that contains an HD-nuclease domain (Makarova et al., 2020; Özcan et al., 2019; Pinilla-Redondo et al., 2019) (Figure S1). Additionally, type IV-A systems encode a CRISPR array and crRNA endonuclease, while type IV-B and type IV-C systems generally do not. It has been proposed that systems lacking a CRISPR array form complexes on crRNAs generated from other CRISPR systems (e.g. type I or type III), but this hypothesis has yet to be explored experimentally. Interestingly, the two subtypes that do not contain a CRISPR array (type IV-B and type IV-C) encode a small α-helical protein (Cas11) predicted to form part of the multi-subunit complex. Thus, there are two distinct type IV multi-subunit complexes, one that contains the small Cas11 subunit (types IV-B and IV-C), and another (type IV-A) that does not contain Cas11 but contains a crRNA derived from a type IV-A CRISPR array and processed by a type IV Cas6 endonuclease. To better understand the function of type IV CRISPR systems as well as their subtype-specific similarities and differences, we isolated a type IV-B complex, analyzed the sequence of the small RNAs bound within the complex, and determined a near-atomic resolution structure.

## Results

### The type IV-B RNP assembles on non-specific RNAs

**Figure 1:**
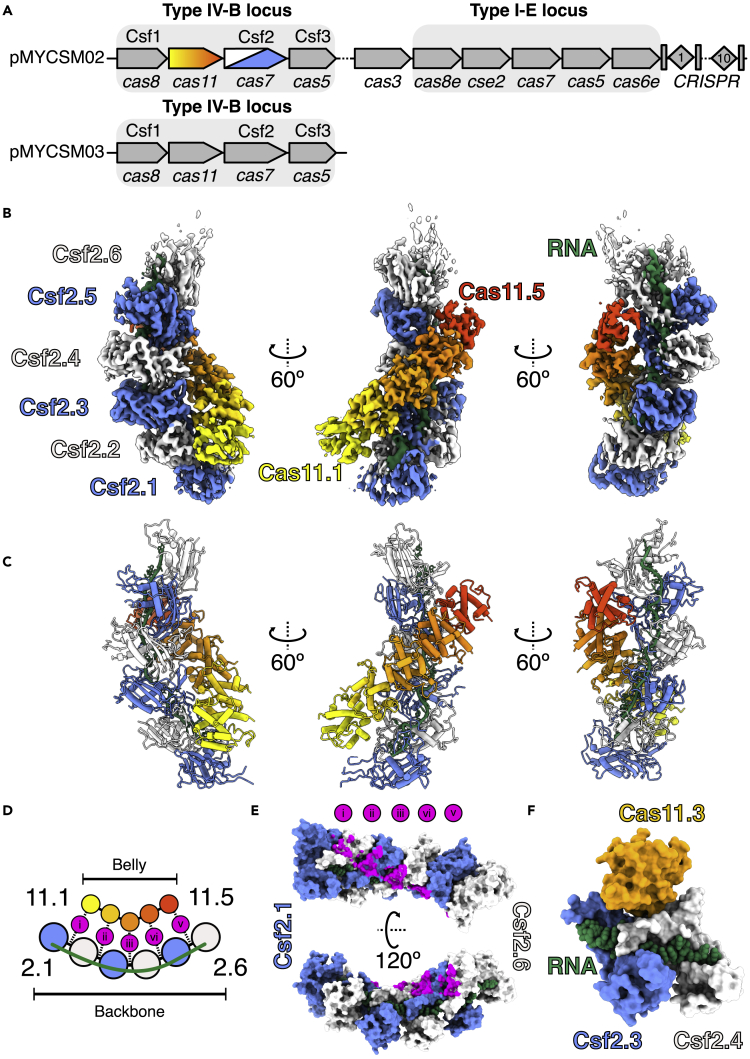
Structure of type IV-B CRISPR complex See also Figures S1–S4 and Table S1. (A) *M. sp*. JS623 plasmid-encoded CRISPR operons. Top: Type IV-B and I-E CRISPR loci present on pMCYCM02 megaplasmid. Bottom: Additional type IV-B locus encoded by pMCYCM03 megaplasmid. Genes predicted to encode RNP complex subunits are indicated with a gray rectangle. (B) 3.9 Å-resolution cryo-EM reconstruction of type IV-B CRISPR complex. Cas7 subunits are colored blue and white, and five Cas11 subunits are colored as a yellow-orange-red gradient. Csf-bound RNA is green. (C) Refined model for the Csf effector complex derived from the cryo-EM maps shown in (B). (D) Schematic of Cas7-Cas11 interactions. Five Csf2-Cas11 interactions occur in this complex (labeled i – v). (E) Positions of Cas11 contacts on Csf2 backbone, colored magenta as shown in panel D. Cas11 sits upon the Csf2-Csf2 interface. (F) Cas11 binds at the interface with buried surface area of 505 Å^2^ (150 Å^2^ and 355 Å^2^ with Csf2.3 and Csf2.4, respectively). Cas11 is completely occluded from bound RNA. Csf2 subunits are intimately connected (1021 Å^2^) and make a network of contacts with bound RNA (∼1200 Å^2^ buried surface area per Csf2 subunit).

The *Mycobacterium sp*. JS623 type IV-B CRISPR operon is encoded within a megaplasmid and lacks both a pre-crRNA maturase (Cas6/Csf5 (Özcan et al., 2019, Taylor et al., 2019) and a CRISPR array, containing only *csf1* (Cas8-like large subunit), *cas11* (small subunit), *csf2* (Cas7) and *csf3* (Cas5) genes (Figure 1A). Interestingly, *M. sp. JS623* also harbors a type I-E system (with an associated CRISPR array) on the same megaplasmid, and another type IV-B operon encoded on a different megaplasmid (Figure 1A), suggesting that type IV-B complexes may assemble on crRNAs encoded and processed by other CRISPR systems. However, the structure and function of such hybrid complexes are unknown.

To gain mechanistic insights into the type IV-B system, we transformed *E. coli* BL21 cells with an expression plasmid encoding the *M. sp.* JS623 type IV-B Cas proteins, and the *M. sp.* JS623 type I-E Cas6 and associated CRISPR array (Figure S2A). Using strep-tag affinity, size exclusion chromatography, and subsequent negative stain we observed filamentous RNP complexes that eluted close to the void volume and a smaller, discrete, RNA-containing species reminiscent of class 1 multi-subunit crRNA-guided complexes (Figure S2) (Makarova et al., 2017). While this latter fraction contained all four Csf subunits, Csf2 and Cas11 were the most abundant (Figure S2). Despite the appearance of a uniform band length of ∼55–60 nucleotides on denaturing PAGE (Figures S2D and S3A), RNAseq analysis revealed bound RNAs were heterogeneous in sequence identity. Few RNAs were derived from the plasmid-encoded CRISPR array, while the majority of Csf-bound RNAs originated from the expression plasmid (63%) (Figures S3B and S3C). To exclude the possibility that this was due to low expression of the CRISPR array and/or lack of crRNA processing by Cas6, we repeated this analysis and compared it to an RNA-seq analysis of the total cellular population of RNAs (total RNA) extracted from the same host (Figure S3D). These results showed that the CRISPR array was indeed expressed and processed by Cas6, resulting in mature crRNAs with a typical eight nucleotide 5′ handle (a characteristic for Cas6-mediated cleavages in the repeats). However, the mature crRNAs were not enriched in the RNAs isolated from type IV RNPs and were in low abundance (∼0.12% of all reads). The apparent lack of sequence specific assembly of the Csf complex on mostly non-crRNAs is different from other CRISPR-Cas systems (Makarova et al., 2017), and might be indicative of a role of type IV CRISPR-Cas systems in functions other than antiviral defense.

### The architecture of the type IV-B RNP resembles type III effector complexes

To compare the type IV-B RNP complex to the complexes of other class 1 systems, we next determined a cryo-EM structure of the IV-B Csf complex at 3.9 Å resolution (Figures 1B and S4, Table S1), allowing us to build an atomic model of the complex *de novo* (Figure 1C). The type IV-B complex resembles a sea cucumber, with six Csf2 (Cas7-like) subunits forming a helical “backbone,” and five Cas11 subunits comprising a helical “belly”. Each Cas11 subunit sits upon a Csf2-Csf2 interface (Figures 1D–1F). The “α-helix bundle” topology of Cas11 (Figure S5C) and presence of a contiguous positively-charged patch running along the length of the minor filament (Figure S6) are typical of Cas11 small subunits in class 1 CRISPR systems (Rollins et al., 2019; Xiao et al., 2017), although the arrangement of helices within type IV Cas11 is distinct from type I and type III small subunits.

**Figure 2:**
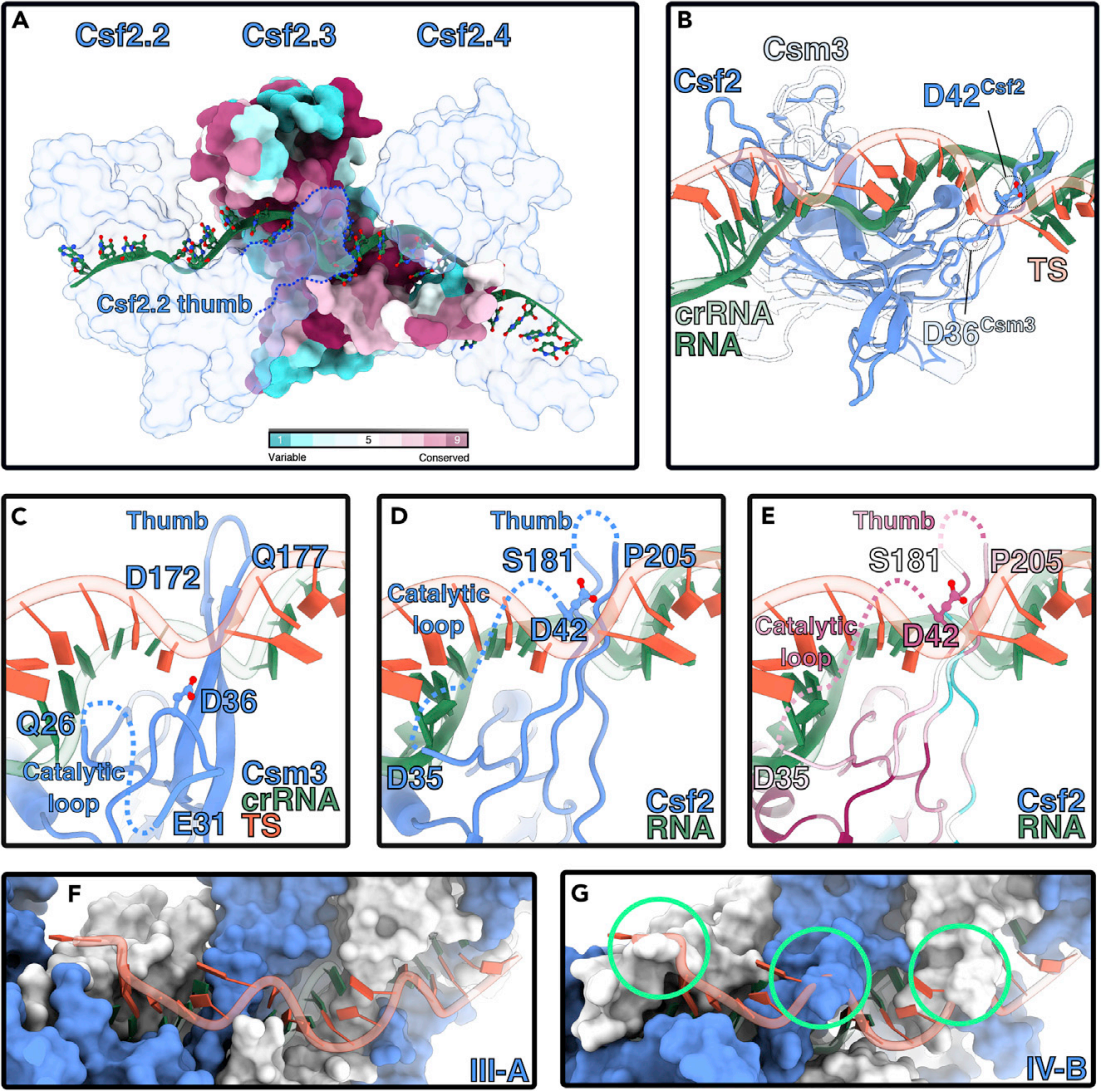
RNA-binding by type IV-B Cas7 See also Figures S5–S7. (A) RNA (green) binding site runs across the palms of Csf2 subunits. Csf2.3 is colored according to conservation. The “thumb” of the n_-1_ Csf2 (i.e. Csf2.2) protrudes into the backbone of bound RNA (solid green), inducing a kink. (B) Alignment of type III-A backbone subunit Csm3 (PDB 607i, transparent) with Csf2 (solid blue). Csm3 and Csf2 align with an r.m.s.d. of 2.9Å, with a Dali server Dali server *Z* score 14.1. Csf2-bound RNA binds in the same conformation as crRNA (transparent green) to Csm3 (RMSD of 1.5 Å). Catalytic residue Asp36^Csm3^ and putative catalytic residue Asp42^Csf2^ side chains are located near the target strand (TS - transparent red), bound to the type III crRNA (transparent green). (C) Residues flanking unstructured catalytic loop (27–35) and apical loop of Csm3 thumb also interact with the TS. Catalytic residue D36 is shown for clarity. (D) Putative interactions with Csf2 and TS, based on alignments with the Csm complex. (E) Putative interactions colored by conservation. The Csf2 thumb contains a flexible 20 residue insertion, not visible in our cryo-EM map. (F), Path of TS bound by type III-A Csm complex. (G) Putative path of TS along IV-B. Severe classes with TS and Csf2 are circled in green.

Like other class 1 Cas7 proteins, Csf2 adopts a hand-shaped structure with fingers, a palm, and a thumb. The palm makes extensive contacts with the bound RNA (buried surface area of ∼1200 Å^2^ per Csf2 subunit) (Figure 2A), while the thumbs of neighboring Csf2 subunits protrude into the center of the palm, inducing a kink in the RNA backbone and a “flipped” base at six nucleotide intervals (typical of other class 1 complexes (Jackson et al., 2014; Taylor et al., 2015)). Using our atomic model of Csf2, we searched for structural homologs. Csf2 had significant similarity to the type III-A CRISPR Csm3 (i.e. Cas7) subunit (Dali *Z* score of 14.1), despite a sequence identity of only 16%. Csf2 and Csm3 superimpose with an r.m.s.d of 2.9 Å and use equivalent interfaces to bind RNA and induce near-identical RNA backbone conformations (r.m.s.d of 1.5 Å) (Figure 2A). This supports previous bioinformatics-based hypotheses that type IV systems originated from type III-like ancestors (Makarova et al., 2020; Özcan et al., 2019; Pinilla-Redondo et al., 2019).

The type III backbone protein Csm3 cleaves the phosphodiester backbone of crRNA-bound target strand (TS) RNA at 6-nt intervals (Staals et al., 2014; Steens et al., 2021). Given that the Csm crRNA aligns almost perfectly with Csf-bound RNA, we reasoned that Csf2 might also possess RNase activity. Within our aligned structures, both the catalytic Asp36^Csm3^ residue and the conserved Asp42^Csf2^ residue are similarly positioned within an unstructured “catalytic loop” (Figures 2C–2E and S7). However, despite this similarity, structural alignment with a target-bound type III complex reveals significant steric clashes between the path of the bound nucleic acid target and the Csf2 catalytic loop (Figures 2F and 2G), suggesting a significant conformational rearrangement of subunits would need to occur upon target binding to place = Asp42^Csf2^ in a position amenable to catalyze target RNA cleavage. Thus, additional substrate bound structures and *in vitro* functional assays are needed to more fully explore the possibility of Csf2-mediated RNase activity.

## Discussion

Our structure of the Csf complex provides evidence that type IV-B evolved from type III CRISPR-Cas systems but lost its CRISPR and Cas6-based crRNA processing activity due to functional respecialization. Although the *M. sp*. JS623 type IV-B operon contains both Csf3 (Cas5) and the putative large subunit Csf1, we did not observe corresponding densities within the high-resolution cryo-EM structure. However, bands that correspond to Csf1 and Csf3 are observed in SDS-PAGE analysis of the sample (Figure S2D), and there is unmodeled ambiguous density on the top and bottom of the complex that could represent a flexible association with Csf1 and Csf3 or additional Csf2 subunits. In type I CRISPR systems, Cas5 binds the 5′ crRNA handle with high affinity and sequence specificity, nucleating complex assembly (Chowdhury et al., 2017; Hochstrasser et al., 2016; Jia et al., 2019). The lack of discernible density for the Cas5-like Csf3 subunit within our complex may explain the heterogeneous assembly of type IV-B Csf complexes around non-specific RNA (Figure S3). However, because the type IV-B system does not encode a CRISPR array, the identity of the RNA sequence that Csf3 would specifically recognize is unknown. Indeed, it remains to be determined whether Csf3 truly serves a similar role to the Cas5 subunits in other systems, binding the 5′-handle of processed crRNAs. We hypothesized that crRNAs generated from the adjacent type I-E CRISPR and Cas6 endonuclease would be bound by the type IV-B complex. However, our sequencing analysis showed no enrichment for crRNAs within the RNPs or any other RNAs available in the total sample. Interestingly, recent bioinformatic analysis indicated a negative co-occurrence of type IV-B systems with other CRISPR systems suggesting their function is not dependent on co-occurring CRISPR arrays (Pinilla-Redondo et al., 2019). The ability of the Csf complex to assemble on non-specific RNAs of a uniform length suggests that type IV-B systems may have been functionally repurposed for a yet to be identified role.

The lack of discernible density for the Csf3 and Csf1 subunits suggests our structure may not accurately reflect the functional type IV-B Csf effector complex. However, several lines of reasoning argue that even without obvious density for Csf1 and Csf3, this complex provides important insights into understanding type IV-B system function. Superposition of the helical Cas7 backbones from type III effector complexes with our structure shows that they are nearly identical in arrangement (Figure S5A). Additionally, the crRNA from the type IV RNP can be overlaid on that of the type III effector with an r.m.s.d. of 1.5 Å (Figure 2A), indicating our complex presents RNA in a conformation amenable for base pairing with complementary nucleic acid. In fact, studies have shown that there are no structural differences between filaments assembled around non-specific RNAs and correctly processed crRNAs bound to the effector (Hochstrasser et al., 2016). Importantly, the structures of all CRISPR-Cas effector complexes involve non-sequence specific interactions between the crRNA and Cas7-like backbone proteins, suggesting that there would be no structural differences between a random RNA and a crRNA bound within the Cas7 backbone of an RNP complex. Thus, our structure likely accurately represents the structure of the Cas7-like core of the effector complex even though it is bound to heterogeneous RNA, and no density is observed for Csf1 and Csf3. Completely novel information is gleaned from our cryo-EM reconstruction of the type IV-B RNP including (1) the first structure of a type IV Cas11 protein, which adopts a novel small subunit fold, (2) the first structure of a Cas7-like Csf2 subunit, and (3) interactions between these subunits with each other and with bound RNA.

Since all type IV systems identified lack adaptation subunits and almost all (97.8%) type IV-B operons identified lack a CRISPR array, it is likely they do not participate in selective pre-spacer acquisition or adaptive immunity (Makarova et al., 2020; Özcan et al., 2019; Pinilla-Redondo et al., 2019). Instead, they may have been co-opted for an orthogonal function. While there is a precedent for the repurposing of CRISPR systems for non-defense functions (Halpin-Healy et al., 2020; Klompe et al., 2019), the role of type IV-B systems remains a mystery. A particularly tantalizing hypothesis is that type IV-B Csf complexes assemble on small RNAs, acting as non-specific RNA-sponges, and enabling IV-B-encoding megaplasmids to evade targeting by host cell RNA guided defenses (Pinilla-Redondo et al., 2019). Future experiments are essential to reveal the biological functions of type IV systems. Recent classifications have indicated that although type IV-B systems are highly diverse, they are almost always associated with an adenosine 5′-phosphosulfate reductase-family gene *cysH* (Makarova et al., 2020; Özcan et al., 2019; Pinilla-Redondo et al., 2019) (Figure S1). Thus, understanding the interplay between *cysH* and the type IV-B Csf RNP complex may be the key to deciphering the enigmatic role of type IV-B CRISPR systems.

### Limitations of the study

The current structure lacks discernible density for Csf1 and Csf3 proteins. The equivalent subunits in Type I systems are responsible for specific functions. Without complementary functional *in vitro* and *in vivo* data, it is impossible to unambiguously characterize the current structure as a functional effector complex.

### Resource availability

#### Lead contact

Further information and requests for resources and reagents should be directed to and will be fulfilled by the lead contact, David W. Taylor (dtaylor@utexas.edu).

#### Materials availability

All unique/stable reagents generated in this study are available from the lead contact without restriction.

#### Data and code availability

The cryo-EM structure and associated atomic coordinates have been deposited in the Electron Microscopy DataBank and the Protein DataBank with accession codes EMD-22340 and PDB: 7JHY, respectively. The accession number for the RNA sequencing data reported in this paper is SRA: SUB8825456.

## Methods

All methods can be found in the accompanying Transparent methods supplemental file.

## Supplemental information

Document S1. Transparent methods, Figures S1–S7, and Table S1
