# Supplementary material for: Structure of a type IV CRISPR-Cas ribonucleoprotein complex
Source: iScience. 2021 Feb 17;24(3):102201. doi: 10.1016/j.isci.2021.102201 (PMC7937560; doi:10.1016/j.isci.2021.102201)
Supplement: Document S1. Transparent methods, Figures S1–S7, and Table S1 [file mmc1.pdf]

iScience, Volume *24*

Supplemental information

Structure of a type IV CRISPR-Cas ribonucleoprotein complex

Yi Zhou, Jack P.K. Bravo, Hannah N. Taylor, Jurre A. Steens, Ryan N. Jackson, Raymond H.J. Staals, and David W. Taylor

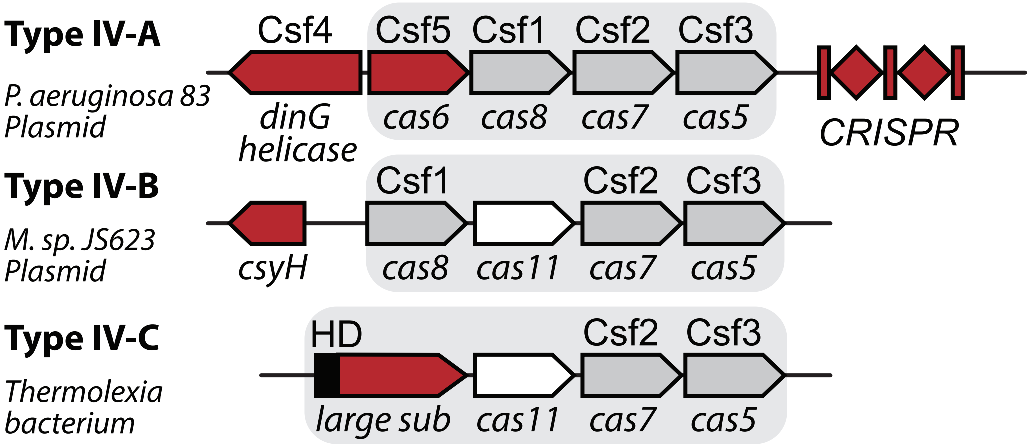

Figure S1. Classification of type IV subtypes. Related to Figure 1. Schematic of the three distinct type IV subtypes defined in (Makarova et al., 2020). Genetic features (genes and CRISPRs) found primarily in a single subtype are colored red. Gray rectangles indicate genes expected to encode proteins that form RNP complexes. The *cas11* gene is colored white to highlight that it is found within the subtypes lacking a CRISPR (subtypes IV-B and IV-C). The HD nuclease domain of the cas8-like subunit of IV-C is colored black.

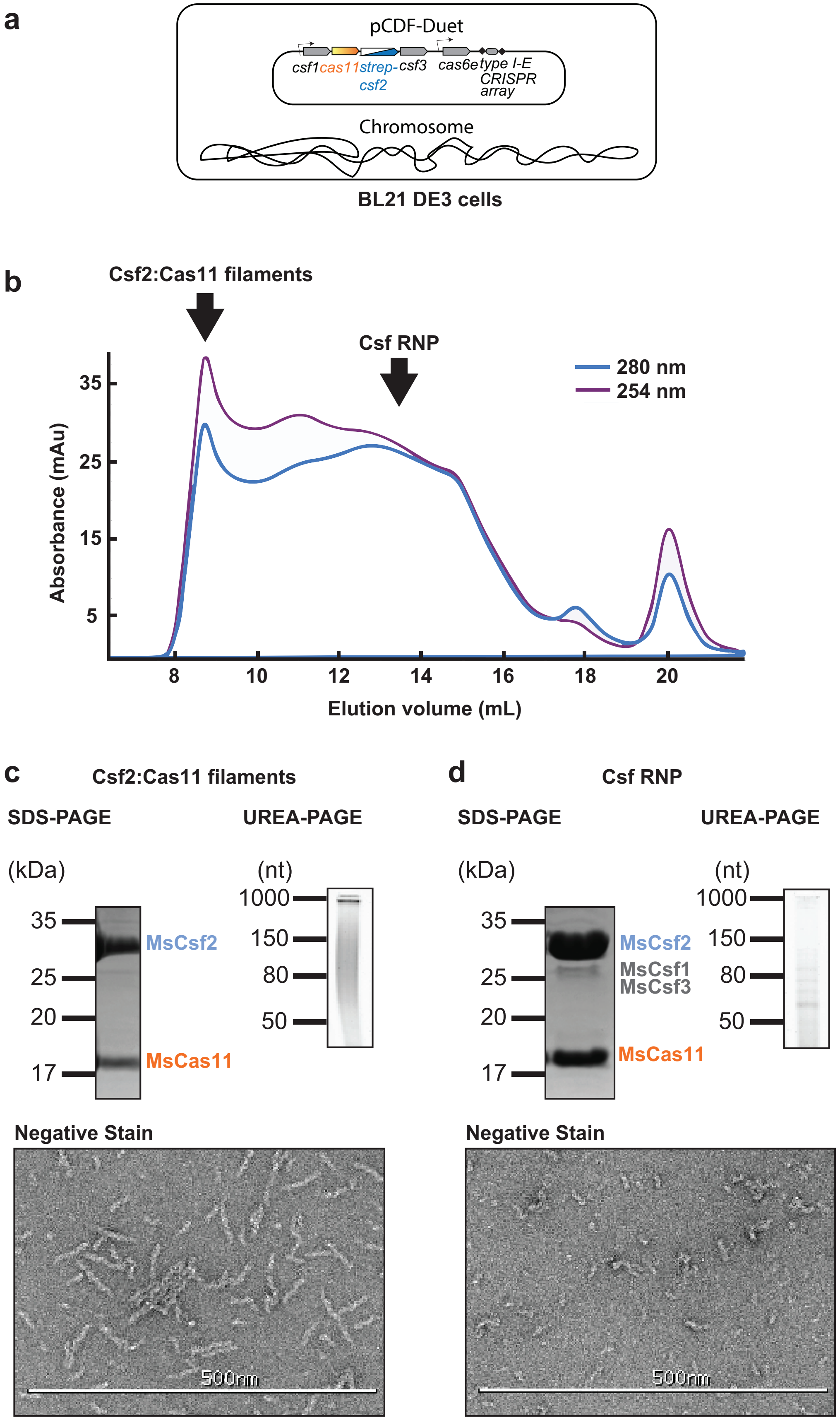
a
pCDF-Duet
csf1 cas11 strep-csf2 csf3 cas6e type I-E CRISPR array
Chromosome
BL21 DE3 cells
b
Csf2:Cas11 filaments
Csf RNP
280 nm
254 nm
Absorbance (mAu)
35
25
15
5
8
10
12
14
16
18
20
Elution volume (mL)
c
Csf2:Cas11 filaments
SDS-PAGE
UREA-PAGE
(kDa)
35
25
20
17
MsCsf2
MsCas11
(nt)
1000
150
80
50
d
Csf RNP
SDS-PAGE
UREA-PAGE
(kDa)
35
25
20
17
MsCsf2
MsCsf1
MsCsf3
MsCas11
(nt)
1000
150
80
50
Negative Stain
500nm
Negative Stain
500nm

Figure S2. Purification of the *M. sp JS623* RNP complex by affinity chromatography (N-Strep-MsCsf2) and size exclusion chromatography. Related to Figure 1. a, Diagram of plasmid used to express the type IV-B complex in BL21 DE3 cells. **b,** SEC chromatogram highlighting peaks corresponding to Csf2:Cas11 filaments and the RNP complex. **c,** SDS-PAGE, UREA-PAGE, and negative stain data indicating the presence of Csf2:Cas11 bound to long RNAs to create filamentous structures. **d,** SDS-PAGE, UREA-PAGE, and negative stain data indicating the presence of Csf1, Csf2, Csf3, and Cas11 bound to short, distinct RNAs to create an RNP complex.

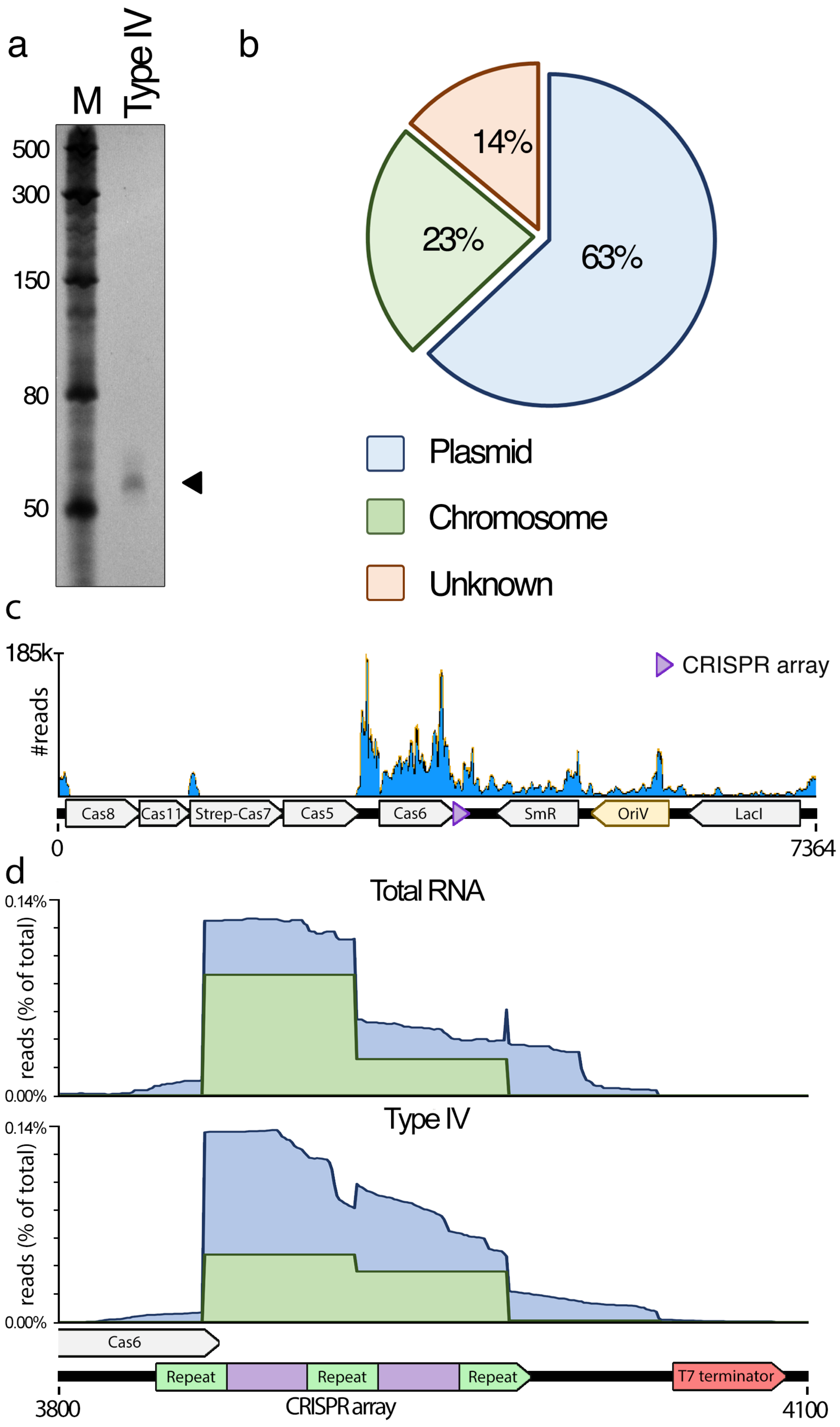

a
M
Type IV
500
300
150
80
50
b
14%
23%
63%
Plasmid
Chromosome
Unknown
c
185k
#reads
CRISPR array
Cas8
Cas11
Strep-Cas7
Cas5
Cas6
SmR
OriV
LacI
0
7364
d
Total RNA
0.14%
0.00%
reads (% of total)
Type IV
0.14%
0.00%
reads (% of total)
Cas6
Repeat
Repeat
Repeat
T7 terminator
3800
CRISPR array
4100

Figure S3. RNA sequencing on co-purifying nucleotides with type IV complex. Related to Figure 1. a, UREA-PAGE gel showing nucleic acids co-purifying with the type IV-B complex. The triangle indicates the ~60nt band purified from isolated type IV-B complexes that was used for RNA sequencing analysis. **b,** Percentage of reads mapping to either to expression plasmid, chromosome, or of unknown origin. **c,** Distribution of reads mapping on the expression plasmid. **d**, Comparison of repeat-containing RNAs from the total cellular RNA population ("total RNA") and type IV RNP-associated ("type IV") RNAs mapped on the CRISPR array of the expression plasmid (in blue). Reads indicated in green represent the 61-nt RNAs with a perfect repeat-derived 8-nt 5' handle, reminiscent of Cas6-mediated cleavages in the repeats.

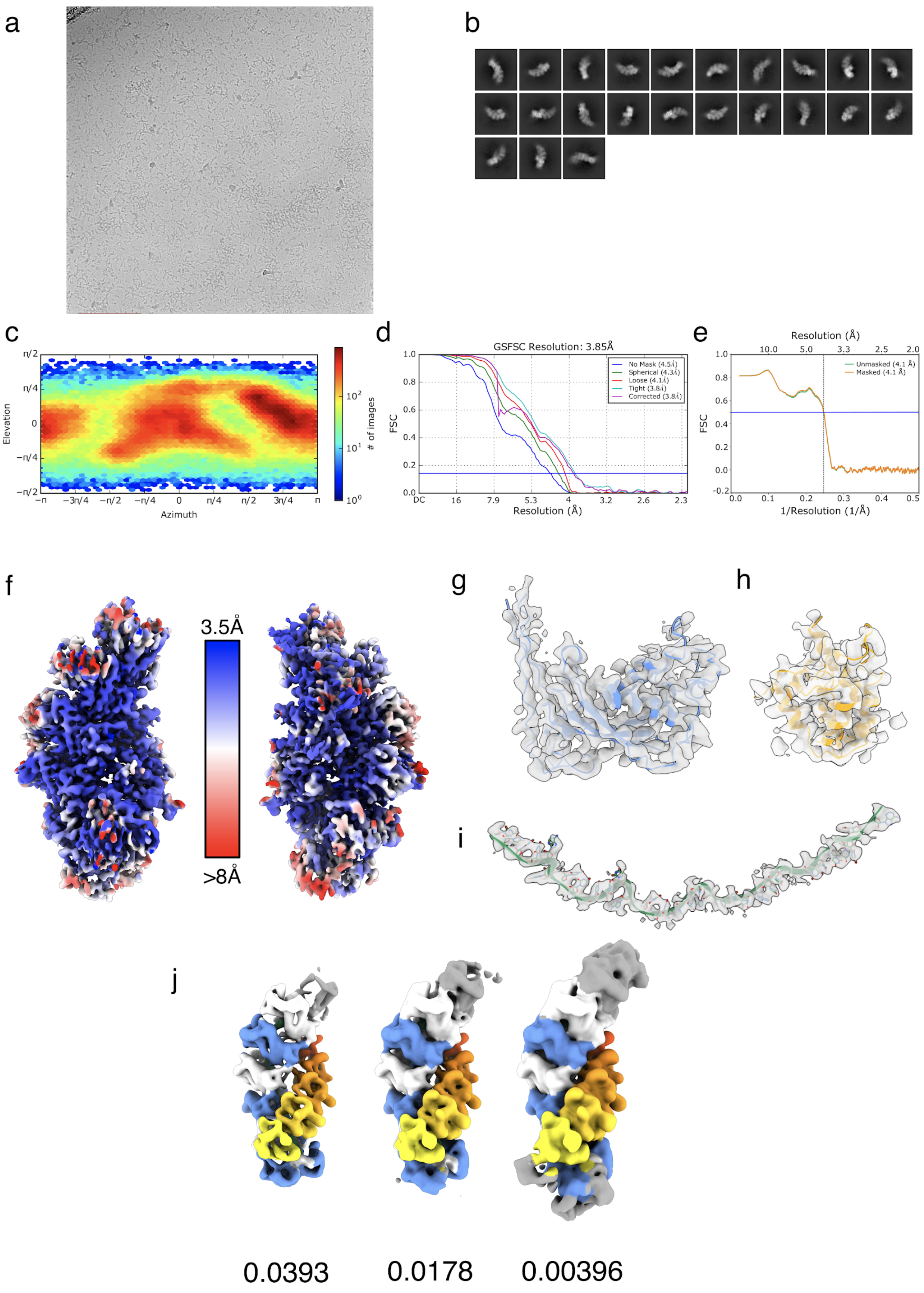
a
b
c
π/2
π/4
0
−π/4
−π/2
Elevation
−π
−3π/4
−π/2
−π/4
0
π/4
π/2
3π/4
π
Azimuth
10^2
10^1
10^0
# of images
d
GSFSC Resolution: 3.85Å
No Mask (4.5Å)
Spherical (4.3Å)
Loose (4.1Å)
Tight (3.8Å)
Corrected (3.8Å)
FSC
1.0
0.8
0.6
0.4
0.2
0.0
DC
16
7.9
5.3
4
3.2
2.6
2.3
Resolution (Å)
e
Resolution (Å)
10.0
5.0
3.3
2.5
2.0
Unmasked (4.1 Å)
Masked (4.1 Å)
FSC
1.0
0.8
0.6
0.4
0.2
0.0
-0.2
0.0
0.1
0.2
0.3
0.4
0.5
1/Resolution (1/Å)
f
3.5Å
>8Å
g
h
i
j
0.0393
0.0178
0.00396

Figure S4. Cryo-EM analysis of Csf. Related to Figure 1. a, Representative micrograph of Csf complex particles. **b,** 2D class averages of discrete Csf oligomers used for 3D reconstruction. **c,** Euler angular distribution of particles contributing to final 3D reconstruction. **d**, Fourier shell correlation (FSC) of final 3D reconstruction, with a global resolution of 3.9 Å at the 0.143 threshold. **e**, Map-to-model FSC, with a model resolution of 4.1 Å at the 0.5 threshold. **f,** Map of Csf complex colored by local resolution. **g – I,** Representative atomic models and corresponding cryo-EM densities for Csf2 (**g**), Cas11 (**h**) and RNA (**i**). **j,** Low-pass filtered (8 Å) map of Csf complex at three different isosurface thresholds. At lower thresholds (0.0178 & 0.00396), additional density appears at the top of the complex. This density may correspond to Csf1 Csf3 subunits, however the low resolution of this region makes unambiguous subunit assignment impossible.

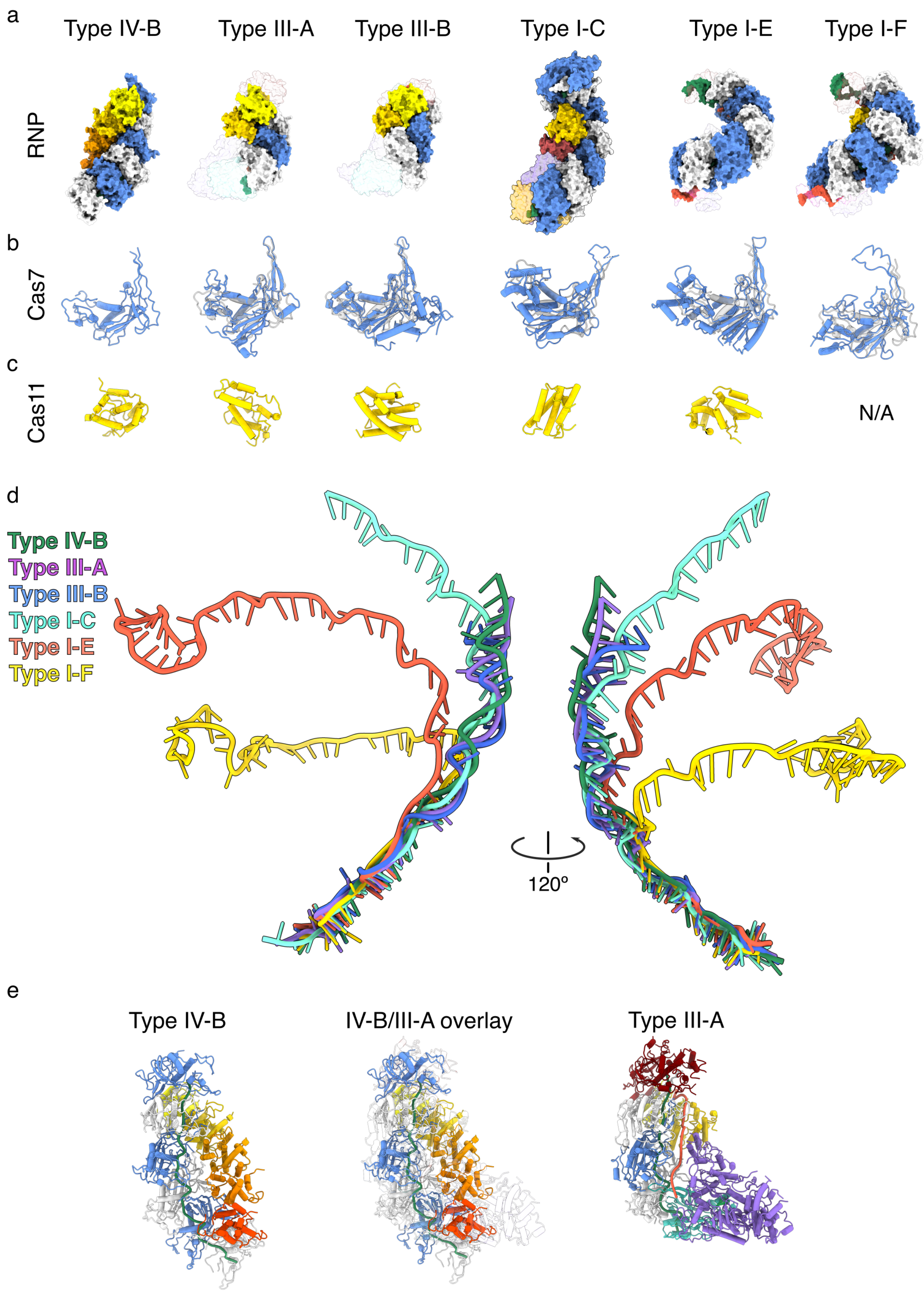
a
Type IV-B
Type III-A
Type III-B
Type I-C
Type I-E
Type I-F
RNP
b
Cas7
c
Cas11
N/A
d
Type IV-B
Type III-A
Type III-B
Type I-C
Type I-E
Type I-F
120º
e
Type IV-B
IV-B/III-A overlay
Type III-A

Figure S5. Comparison to Cascade complexes. Related to Figure 2. a, Comparison of Type IV-B Csf with other effector/Cascade complexes. crRNA within Type III-A (PDB 6o7i) (Jia et al., 2019), III-B (PDB 3x1l) (Osawa et al., 2015) & I-C (O'Brien et al., 2020) (7kha) complexes were aligned to Csf RNA (RMSD 5.9 Å, 8.8 Å, and 12.7 Å respectively). Due to the highly curved nature of type I-E (4tvx) (Jackson et al., 2014) and I-F (5uz9) (Chowdhury et al., 2017) crRNA, it was not possible to perform such alignment to IV-B. Instead, individual backbone subunits were aligned to corresponding Csf2 subunits, thus aligning the top of I-E or I-F with the top of IV-B (RMSD ~24.4 Å and 25.8 Å, respectively). In all complexes, non-Cas7/Cas11 subunits are shown as transparent surfaces. All Cas7/Cas11 and RNA, crRNA and TS are colored as in **Figures 1 & 2**, with the addition of the TS in light red. **b,** Alignment of Cas7 with Csf2. Csf2 is shown as grey, transparent cartoon. RMSD is typically ~20 Å – 25 Å, although they clearly align well by eye. The high RMSD is likely due to presence of additional residues not present in Csf2. **c**, Cas11 subunits. All Cas11 subunits are helical bundles that resemble each other. However, due to diverse Cas11 sequences these subunits align poorly (RMSD 16 Å – 18 Å)**. d,** Alignment of (cr)RNA from available CRISPR effector complexes. Type IV-B RNA aligns more closely to type III-A and -B crRNAs, consistent with the proposed evolutionary lineage of type IV CRISPR systems emerging from a type III-like ancestor. **e,** Overlay of type IV-B and type III-A CRISPR complexes based on RNA alignment. Type III-A displays the strongest structural homology between Cas7 subunits.

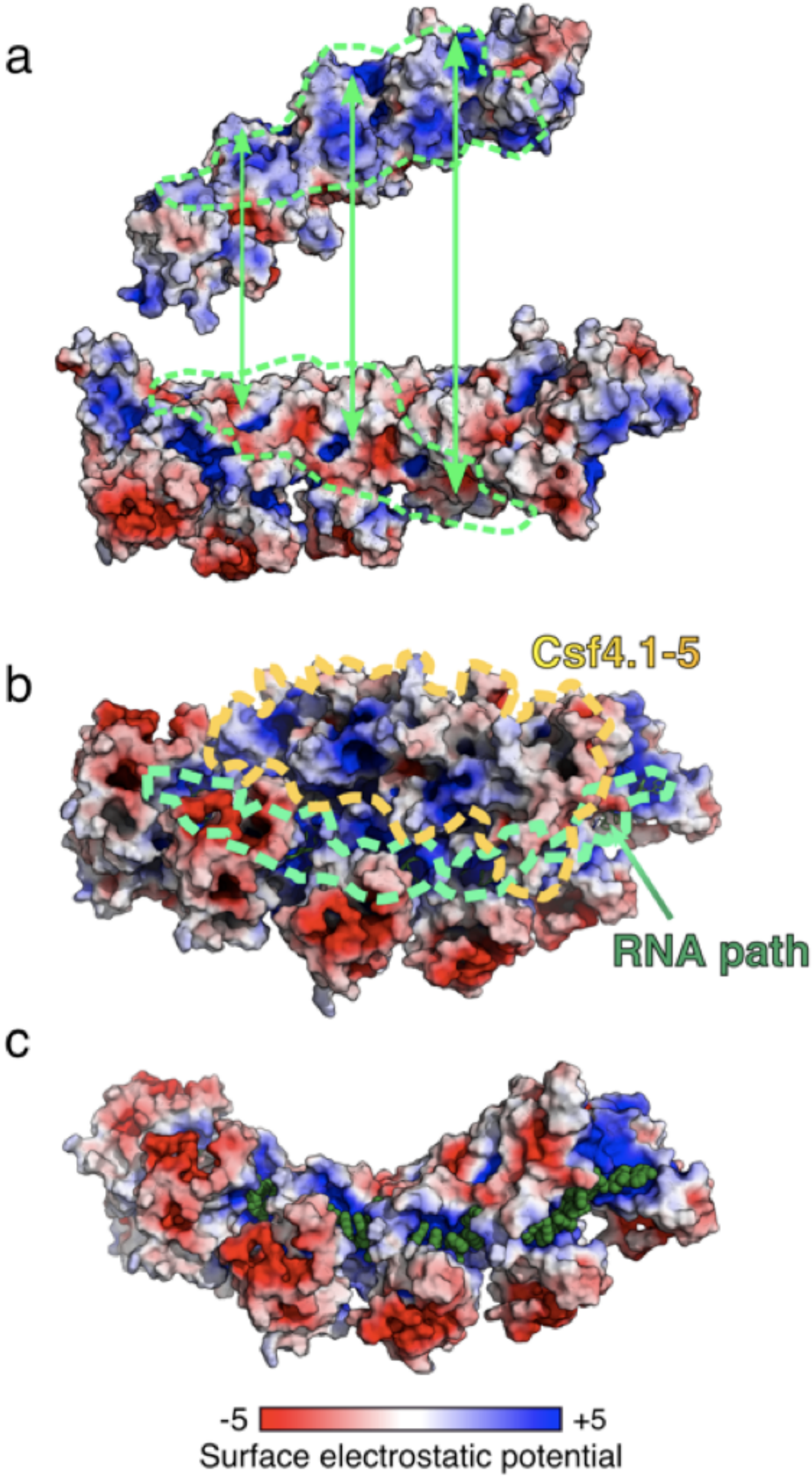
a
b
Csf4.1-5
RNA path
c
-5
+5
Surface electrostatic potential

Figure S6. Surface electrostatics of Csf complex. Related to Figure 2. a & b, Cas11 and Csf2 filaments. Green dashed outlines denote complementary surfaces. **b,** Surface electrostatics of the Csf complex. Cas11 subunits and RNA path are outlined. RNA is shown as green cartoon, but it is almost completely occluded by Cas7 subunits. The overall path of the RNA bound within the Cas7 filament is outlined by green dashed lines. **c,** Csf2 filament with RNA contacts (green spheres) shown. Cas11 minor filament is removed for clarity. RNA is bound by a contiguous positively-charged surface. The high electrostatic contribution to RNA binding by Csf2 is typical of non-specific RNA-binding proteins (Bravo et al., 2020, 2018).

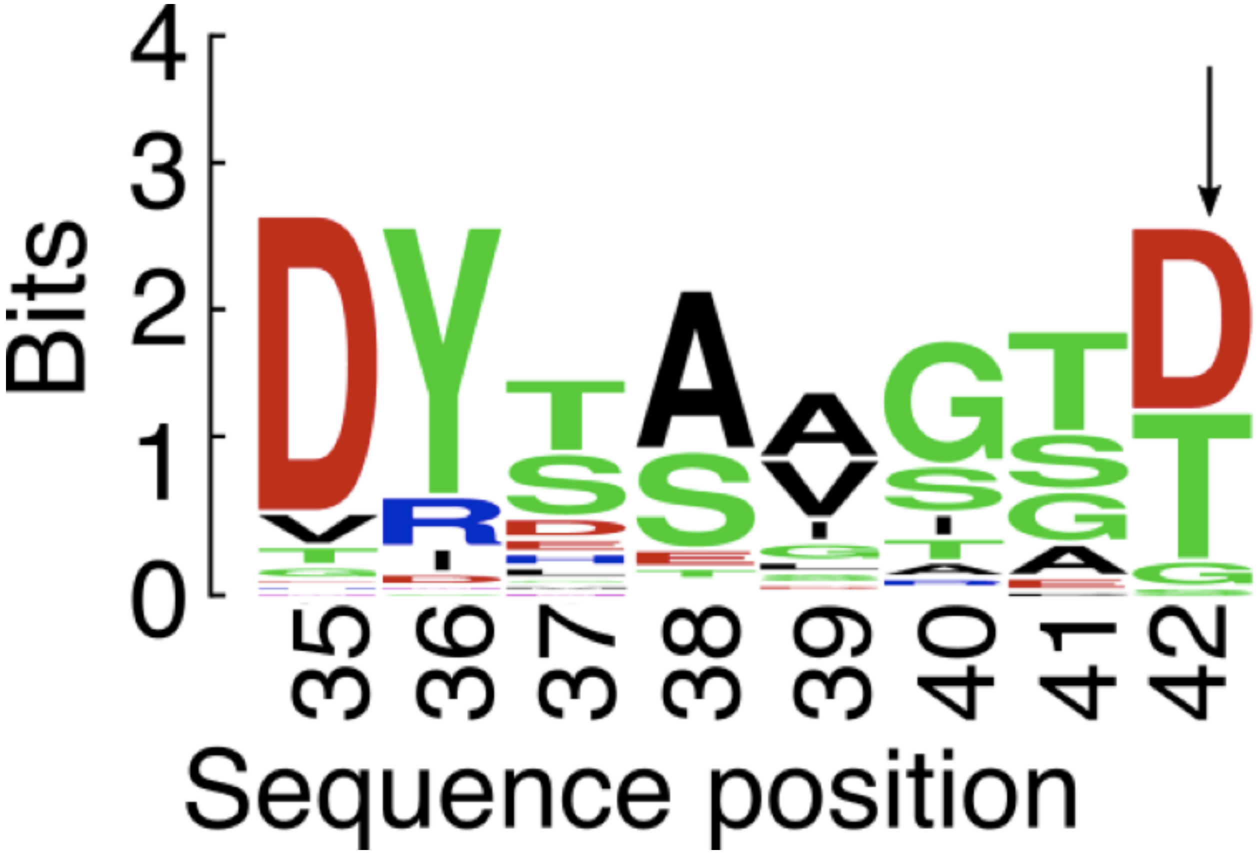

Figure S7. Weblogo of cleavage loop, with candidate catalytic residue (D42) denoted by arrow. Related to Figure 2. Multiple sequence alignment (MSA) generated using the top 100 result from a BLAST search against the Csf2 sequence from *M. sp JS623*. Output from the MSA was used to generate a sequence logo using the WebLogo server (Crooks et al., 2004) and conservation score used in Figure 2.

Data collection and processing	
Magnification	22,500x
Voltage (kV)	300
Electron exposure (e^-/Å)	40
Defocus range (μM)	-1.5 to -3.0
Symmetry imposed	C1
Final particle images	296,319
Map resolution (Å)	3.9
FSC threshold	0.143
Map resolution range (Å)	3.5 to > 8
**Refinement**	
Model resolution (Å) (0.143 FSC)	4.1
Map sharpening *B* factor (Å^2)	130.2
Model composition	
Nonhydrogen atoms	16636
Residues (Protein/RNA)	2083/31
B factors (Å^2) (min/max/mean)	
Protein	19.75/206.87/81.59
RNA	34.71/125.88/56.91
r.m.s. deviations	
Bond lengths (Å)/bond angle (º)	0.005/1.112
**Validation**	
MolProbity score	1.9
Clashscore	4.72
Poor rotamers (%)	0
Ramachandran plot	
Favoured/Allowed/Disallowed (%)	84.99/15.01/0

Table S1. Cryo-EM data collection and processing parameters. Related to Figure 1.

Transparent Methods

Expression and purification of the M. sp JS623 Csf complex

E.coli BL21 (DE3) cells containing the pCDF-Duet1-Csf1-Cas11-Strep-Csf2-Csf3(MCS1)-Cas6 array (MCS2) expression vector were inoculated in 6 X 0.5 L lysogeny broth (LB) and grown at 37°C with 200 rpm shaking. Cells were grown to an optical density (O.D. 600 nm) between 0.6-0.7 then cold shocked on ice for 30-60 min. Recombinant protein expression was induced with 0.8 mM IPTG (isopropyl β-D-1-thiogalactopyranoside). After induction, cells were grown at 16°C for 18-24 hours and pelleted via centrifugation. Pelleted cells were resuspended in 20-30 mL Buffer W (100 mM Tris, pH 8.0; 150 mM NaCl; 2 μM $ZnSO_4$). Protease inhibitors were added to the following final concentrations: 10 μg/mL leupeptin, 2 μg/mL aprotinin, and 170 μg/mL phenylmethylsulfonyl fluoride (PMSF). Cells were lysed by sonication and lysate was clarified by centrifugation. Polyethylenimine was added to the soluble fraction at a final concentration of 0.1% to precipitate nucleic acids and again clarified by centrifugation. The supernatant was applied to a StrepTrap HP 5 mL column (GE Healthcare) and the bound protein was eluted with Buffer E (Buffer W + 5 mL desthiobiotin). The RNP complex was further purified with a Superose6 Increase 10/300 GL column (GE Healthcare), eluting into SEC Buffer (50 mM HEPES, pH 8.0; 150 mM NaCl; 2 μM $ZnSO_4$).

RNA sequencing and analysis

Nucleic acids that co-purified with type IV-B complex were extracted with acid phenol:chloroform and subsequent ethanol precipitation. The resulting fraction was loaded on a 20% denaturing PAGE gel after which a band of approximately 55-60 nt (**Figure S3A**) was excised and purified from gel using the ZR small-RNA PAGE Recovery Kit (Zymo Research, USA). Small RNAs were prepared by GenXPro (GenXPro GmbH, Germany) using the TrueQuant smallRNA Seq kit according to the manufacturer's instructions and were sequenced on a HiSeq2000 (Illumina, USA). After quality control filtering and adapter trimming using Cutadapt (Martin, 2011), the reads were mapped on the expression plasmid and the *E.coli* BL21 (DE3) genome (Genbank accession CP001509) with Geneious Prime 2020.10.2 (https://www.geneious.com). For comparing the abundance and processing of (mature) crRNAs of the total cellular RNA population versus those associated with the type IV complex (Figure S2D), the extracted total RNAs (NEB Monarch Total RNA Miniprep Kit) were first depleted for ribosomal RNAs (Invitrogen Ribominus Transcriptome Isolation Kit) before they were sequenced on an Illumina MiSeq (Center for Integrated Biosystems, Utah State University, USA). After quality control and adapter trimming, the resulting reads were mate-paired and merged using SeqPrep (https://github.com/jstjohn/SeqPrep), filtered for reads containing CRISPR-array repeat nucleotides, and mapped on the expression plasmid using Geneious (Langmead et al., 2009). Visualisation of the mapping results and further downstream analyses were performed using Geneious and Microsoft Excel.

Cryo-EM data acquisition and processing

C-flat holy carbon grids (CF-4/2, Protochips Inc.) were glow-discharged for 30 seconds using a Gatan Solarus plasma cleaner. 2.5 μl of Type IV complex (~0.3 mg/ml) was applied onto grids, blotted for 2.5 seconds with a blotting force of 1 and rapidly plunged into liquid ethane using a FEI Vitrobot MarkIV operated at 4 °C and 100% humidity. Data were acquired using a FEI Titan Krios

transmission electron microscope (Sauer Structural Biology Laboratory, University of Texas at Austin) operating at 300 keV at a nominal magnification of ×22,500 (1.1 Å pixel size) with defocus ranging from -1.5 to -3.0 μm. The data were collected using a total exposure of 6 s fractionated into 20 frames (300 ms per frame) with a dose rate of ~8 electrons per pixel per second and a total exposure dose of ~40 $e^{-}Å^{-2}$. Three datasets were automatically recorded on a Gatan K2 Summit direct electron detector operated in counting mode using the MSI-Template application within the automated macromolecular microscopy software LEGINON (Suloway et al., 2005).

All image pre-processing was performed in Appion (Lander et al., 2009). Individual movie frames were aligned and averaged using 'MotionCor2' drift-correction software (Zheng et al., 2017). The contrast transfer function (CTF) of each micrograph was estimated using CTFFIND4 (Rohou and Grigorieff, 2015). Particles were picked with a template-based particle picker using a reference-free 2D class average from a small subset of manually picked particles as templates. Selected particles were extracted from micrographs using particle extraction within Relion (Scheres, 2012) and the coordinates exported from Appion. After multiple rounds of 2D classification in Relion to remove junk particles, 824,421 particles were left and imported into cryoSPARC (Punjani et al., 2017) for further processing. After multiple rounds of 2D classification and heterogeneous refinement, a final reconstruction containing 296,319 particles was determined to a global resolution of 3.9 Å (based on the gold standard 0.143 FSC criterion using two independent half-maps) using local refinement (implementing non-uniform refinement) with a mask corresponding to the entire complex.

Csf model building, refinement and structural analysis

An atomic model for the Csf complex was built de novo in Coot (Emsley et al, 2004), and subjected to multiple iterative rounds of molecular dynamics - flexible fitting in Namdinator (Kidmose et al, 2019) and real-space refinement in Phenix (Afonine et al., 2018). The majority of the RNA was modelled as polyU, with occasional bases modelled as A depending on the size of the cryoEM density corresponding to the nucleobase (i.e. if the density was unambiguously a purine, (Bravo et al., 2021)). The refined Csf complex model was validated using MolProbity (Chen et al., 2010) as implemented in Phenix. Protein sequence conservation analysis was performed using online ConSurf (Ashkenazy et al., 2016) server, with multiple sequence alignment (MSA) generated using the top 100 result from a BLAST search against Csf sequences. Output from the MSA was used to generate a sequence logo using the WebLogo server (Crooks et al., 2004). Maps and models were visualized using ChimeraX (Goddard et al., 2018) and the electrostatic surfaces were determined using the APBS plugin (Baker et al., 2001) within PyMol. Root-mean-square deviation (r.m.s.d.) values between equivalent atoms in Csf2 and type III-A Csm3, and between type IV-B RNA and III-A crRNA were calculated using ChimeraX and PyMol.

Supplemental References

Afonine, P. V., Poon, B.K., Read, R.J., Sobolev, O. V., Terwilliger, T.C., Urzhumtsev, A., Adams, P.D., 2018. Real-space refinement in PHENIX for cryo-EM and crystallography. Acta Crystallogr. Sect. D Struct. Biol. 74, 531–544.

Ashkenazy, H., Abadi, S., Martz, E., Chay, O., Mayrose, I., Pupko, T., Ben-Tal, N., 2016. ConSurf 2016: an improved methodology to estimate and visualize evolutionary conservation in macromolecules. Nucleic Acids Res. 44, W344–W350.

Baker, N.A., Sept, D., Joseph, S., Holst, M.J., McCammon, J.A., 2001. Electrostatics of nanosystems: Application to microtubules and the ribosome. Proc. Natl. Acad. Sci. U. S. A. 98, 10037–10041.

Bravo, J.P.K., Bartnik, K., Venditti, L., Gail, E.H., Davidovich, C., Lamb, D.C., Tuma, R., Calabrese, A.N., Borodavka, A., 2020. Structural basis of rotavirus RNA chaperone displacement and RNA annealing. bioRxiv 2020.10.26.354233.

Bravo, J.P.K., Borodavka, A., Barth, A., Calabrese, A.N., Mojzes, P., Cockburn, J.J.B., Lamb, D.C., Tuma, R., 2018. Stability of local secondary structure determines selectivity of viral RNA chaperones. Nucleic Acids Res. 293191.

Bravo, J.P.K., Dangerfield, T.L., Taylor, D.W., Johnson, K.A., 2021. Remdesivir is a delayed translocation inhibitor of SARS CoV-2 replication. Mol. Cell.

Chen, V.B., Arendall, W.B., Headd, J.J., Keedy, D.A., Immormino, R.M., Kapral, G.J., Murray, L.W., Richardson, J.S., Richardson, D.C., 2010. MolProbity: All-atom structure validation for macromolecular crystallography. Acta Crystallogr. Sect. D Biol. Crystallogr. 66, 12–21.

Chowdhury, S., Carter, J., Rollins, M.C.F., Golden, S.M., Jackson, R.N., Hoffmann, C., Nosaka, L., Bondy-Denomy, J., Maxwell, K.L., Davidson, A.R., Fischer, E.R., Lander, G.C., Wiedenheft, B., 2017. Structure Reveals Mechanisms of Viral Suppressors that Intercept a CRISPR RNA-Guided Surveillance Complex. Cell 169, 47-57.e11.

Crooks, G., Hon, G., Chandonia, J., Brenner, S., 2004. WebLogo: a sequence logo generator. Genome Res 14, 1188–1190.

Goddard, T.D., Huang, C.C., Meng, E.C., Pettersen, E.F., Couch, G.S., Morris, J.H., Ferrin, T.E., 2018. UCSF ChimeraX: Meeting modern challenges in visualization and analysis. Protein Sci. 27, 14–25.

Jackson, R.N., Golden, S.M., van Erp, P.B.G., Carter, J., Westra, E.R., Brouns, S.J.J., van der Oost, J., Terwilliger, T.C., Read, R.J., Wiedenheft, B., 2014. Structural biology. Crystal structure of the CRISPR RNA-guided surveillance complex from Escherichia coli. Science 345, 1473–1479.

Jia, N., Mo, C.Y., Wang, C., Eng, E.T., Marraffini, L.A., Patel, D.J., 2019. Type III-A CRISPR-Cas Csm Complexes: Assembly, Periodic RNA Cleavage, DNase Activity Regulation, and Autoimmunity. Mol. Cell 73, 264-277.e5.

Lander, G.C., Stagg, S.M., Voss, N.R., Cheng, A., Fellmann, D., Pulokas, J., Yoshioka, C.,

Irving, C., Mulder, A., Lau, P.W., Lyumkis, D., Potter, C.S., Carragher, B., 2009. Appion: An integrated, database-driven pipeline to facilitate EM image processing. J. Struct. Biol. 166, 95–102.

Langmead, B., Trapnell, C., Pop, M., Salzberg, S.L., 2009. Ultrafast and memory-efficient alignment of short DNA sequences to the human genome. Genome Biol. 10.

Makarova, K.S., Wolf, Y.I., Iranzo, J., Shmakov, S.A., Alkhnbashi, O.S., Brouns, S.J.J., Charpentier, E., Cheng, D., Haft, D.H., Horvath, P., Moineau, S., Mojica, F.J.M., Scott, D., Shah, S.A., Siksnys, V., Terns, M.P., Venclovas, Č., White, M.F., Yakunin, A.F., Yan, W., Zhang, F., Garrett, R.A., Backofen, R., van der Oost, J., Barrangou, R., Koonin, E. V., 2020. Evolutionary classification of CRISPR–Cas systems: a burst of class 2 and derived variants. Nat. Rev. Microbiol. 18, 67–83.

Martin, M., 2011. Cutadapt removes adapter sequences from high-throughput sequencing reads. EMBnet.journal [Online] 17, 10–12.

O'Brien, R.E., Santos, I.C., Wrapp, D., Taylor, D.W., Bravo, J.P.K., Schwartz, E.A., Brodbelt, J.S., 2020. Structural basis for assembly of non-canonical small subunits into type I-C Cascade. Nat. Commun. 1–6.

Osawa, T., Inanaga, H., Sato, C., Numata, T., 2015. Crystal structure of the crispr-cas RNA silencing cmr complex bound to a target analog. Mol. Cell 58, 418–430.

Punjani, A., Rubinstein, J.L., Fleet, D.J., Brubaker, M.A., 2017. CryoSPARC: Algorithms for rapid unsupervised cryo-EM structure determination. Nat. Methods 14, 290–296.

Rohou, A., Grigorieff, N., 2015. CTFFIND4: Fast and accurate defocus estimation from electron micrographs. J. Struct. Biol. 192, 216–221.

Scheres, S.H.W., 2012. RELION: Implementation of a Bayesian approach to cryo-EM structure determination. J. Struct. Biol. 180, 519–530.

Suloway, C., Pulokas, J., Fellmann, D., Cheng, A., Guerra, F., Quispe, J., Stagg, S., Potter, C.S., Carragher, B., 2005. Automated molecular microscopy: The new Leginon system. J. Struct. Biol. 151, 41–60.

Zheng, S.Q., Palovcak, E., Armache, J.P., Verba, K.A., Cheng, Y., Agard, D.A., 2017. MotionCor2: Anisotropic correction of beam-induced motion for improved cryo-electron microscopy. Nat. Methods 14, 331–332.
